# β-Hairpin Alignment Alters Oligomer Formation in Aβ-Derived Peptides

**DOI:** 10.1021/acs.biochem.3c00526

**Published:** 2024-01-01

**Authors:** Sarah M. Ruttenberg, Adam G. Kreutzer, Nicholas L. Truex, James S. Nowick

**Affiliations:** Department of Chemistry, University of California, Irvine Irvine, California 92697-2025, United States

## Abstract

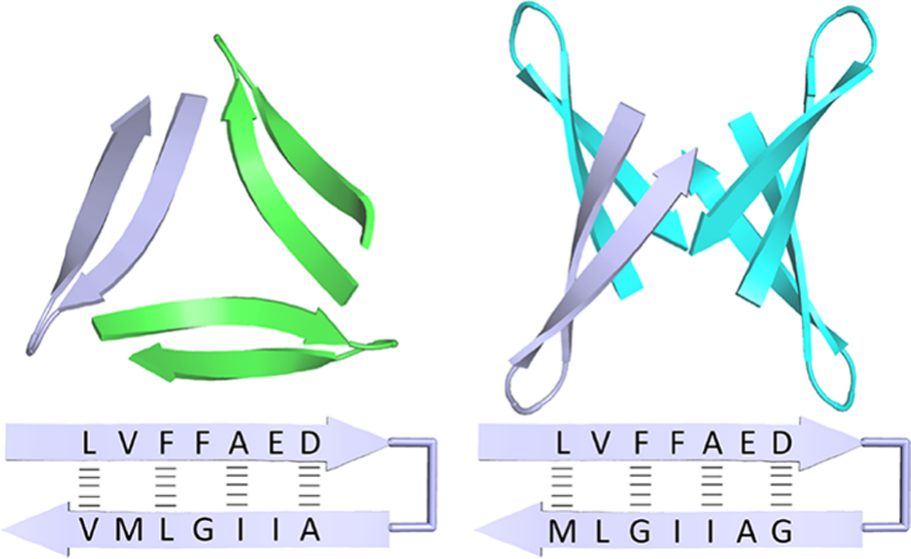

Amyloid-β (Aβ) forms heterogeneous oligomers, which are implicated in the pathogenesis of Alzheimer’s disease (AD). Many Aβ oligomers consist of β-hairpin building blocks—Aβ peptides in β-hairpin conformations. β-Hairpins of Aβ can adopt a variety of alignments, but the role that β-hairpin alignment plays in the formation and heterogeneity of Aβ oligomers is poorly understood. To explore the effect of β-hairpin alignment on the oligomerization of Aβ peptides, we designed and studied two model peptides with two different β-hairpin alignments. Peptides Aβm_17–36_ and Aβm_17–35_ mimic two different β-hairpins that Aβ can form, the Aβ_17–36_ and Aβ_17–35_ β-hairpins, respectively. These hairpins are similar in composition but differ in hairpin alignment, altering the facial arrangements of the side chains of the residues that they contain. X-ray crystallography and SDS-PAGE demonstrate that the difference in facial arrangement between these peptides leads to distinct oligomer formation. In the crystal state, Aβm_17–36_ forms triangular trimers that further assemble to form hexamers, while Aβm_17–35_ forms tetrameric β-barrels. In SDS-PAGE, Aβm_17–36_ assembles to form a ladder of oligomers, while Aβm_17–35_ either assembles to form a dimer or does not assemble at all. The differences in the behavior of Aβm_17–36_ and Aβm_17–35_ suggest β-hairpin alignment as a source of the observed heterogeneity of Aβ oligomers.

## Introduction

Amyloid-β (Aβ) is intrinsically disordered and can adopt myriad conformations, including β-sheets, α-helices, and a variety of β-hairpins.^1−18^ These secondary structures can direct the formation of toxic Aβ assemblies implicated in Alzheimer’s disease (AD).^4,6,9,11,12,15−18^ Several studies have demonstrated that toxic Aβ oligomers consist of β-hairpin building blocks.^4,6,9,12,19^ Multiple β-hairpins have been reported for Aβ that differ in the alignment of the β-strands and the residues they contain.^1,4,6−13,18,19^ The role that β-hairpin alignment plays in the formation and heterogeneity of oligomers of Aβ is poorly understood. Atomic-level analysis of these oligomers is necessary to better understand the molecular basis of AD, but the transience of Aβ oligomers makes their characterization difficult.

To better study these elusive Aβ oligomers, our laboratory has developed peptide model systems consisting of conformationally constrained β-hairpin peptides derived from Aβ.^20−27^ These model systems have provided a variety of high-resolution structures of oligomeric assemblies that cannot be achieved from oligomers formed by Aβ itself. In the current study, we use two similar model peptides to explore the effect of β-hairpin alignment on the assembly of peptides derived from Aβ. Aβm_17–36_ and Aβm_17–35_ mimic two different β-hairpins that Aβ can form, the Aβ_17–36_ and Aβ_17–35_ β-hairpins, respectively.^13,18,19^

**Figure 1:**
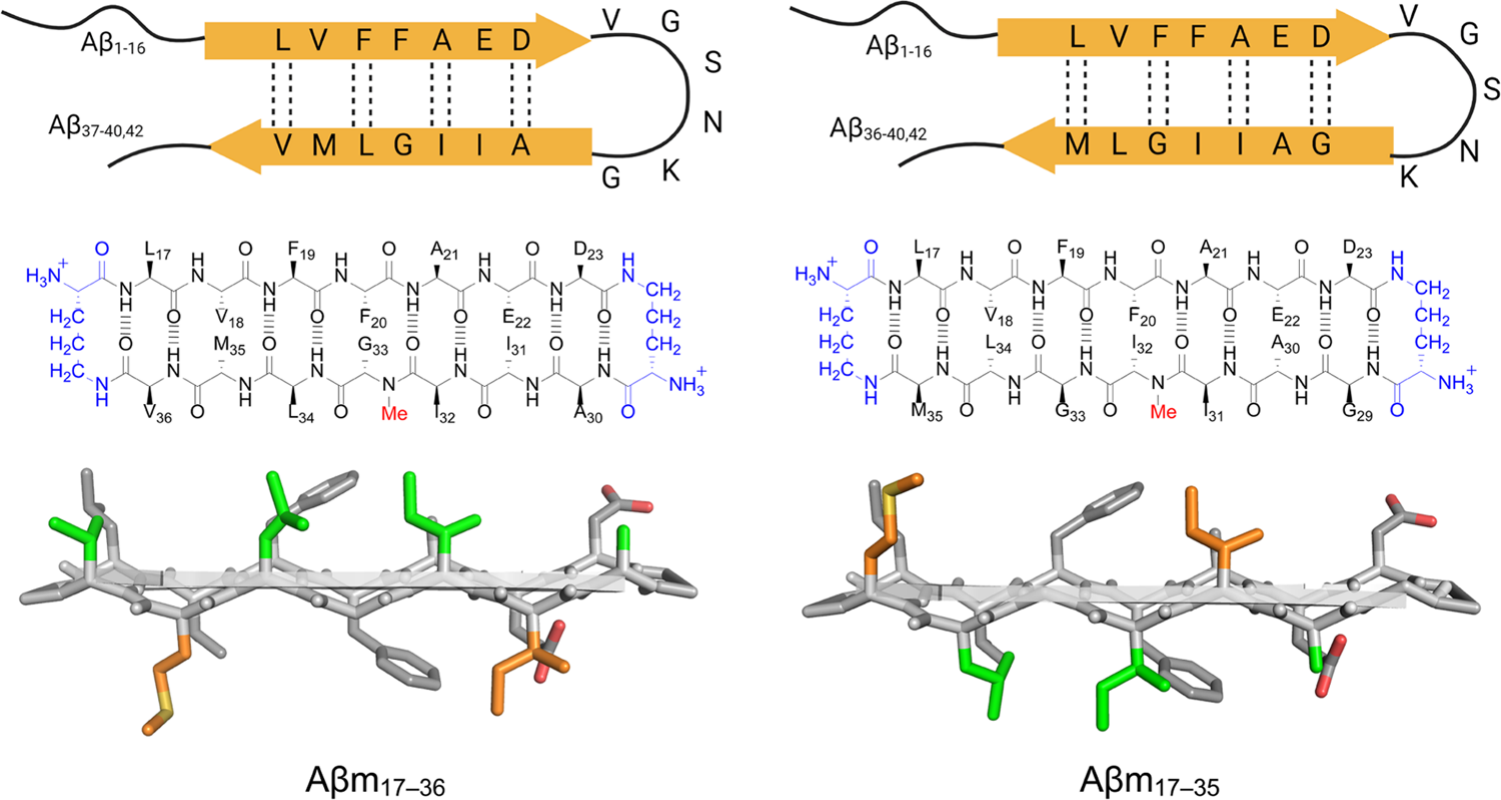
Cartoon representations of the Aβ_17–36_ and Aβ_17–35_ hairpins (top). Chemical drawings (middle) and cartoon representations (bottom) of Aβm_17–36_ and Aβm_17–35_. In the chemical drawings, the δ-linked ornithine turn units are shown in blue, and the *N*-methyl group is shown in red. In the cartoon representations, the residues in Aβm_17–35_ are colored to match those in Aβm_17–36_, indicating the differences between the faces of Aβm_17–36_ and Aβm_17–35_.

Aβm_17–36_ and Aβm_17–35_ differ in the alignment of the β-strands that comprise each peptide. In both peptides, residues 17–23 constitute one of the β-strands. In Aβm_17–36_, residues 30–36 constitute the other β-strand, while in Aβm_17–35_, residues 29–35 constitute the other β-strand (Figure 1). This difference results in a different alignment of the peptide strands, with the bottom strand of Aβm_17–36_ shifted by one amino acid toward the C-terminus in comparison to Aβm_17–35_. Thus, Leu_17_ is across from Val_36_ in Aβm_17–36_, while Leu_17_ is across from Met_35_ in Aβm_17–35_. This shift changes the hydrogen-bonded pairs of the residues within the hairpin as well as the surface on which the side chains are displayed. The change in facial arrangement is illustrated in Figure 1, with the even-numbered side chains (green) displayed on the “top” face in Aβm_17–36_ and the “bottom” face in Aβm_17–35_.

Notably, the chemical compositions of Aβm_17–36_ and Aβm_17–35_ are almost identical—Aβm_17–36_ contains Val_36_ whereas Aβm_17–35_ contains Gly_29_. The similarity of Aβm_17–36_ and Aβm_17–35_ makes them good candidates for exploring how β-hairpin alignment, and thus the facial arrangement of residues, affects oligomeric assembly. In this study, we compare Aβm_17–36_ and Aβm_17–35_ and demonstrate that the alignment of the β-hairpin can affect the assembly of peptides derived from Aβ.

## Results and Discussion

### Design of the Model System

Aβm_17–36_ and Aβm_17–35_ are cyclic hexadecapeptides consisting of two heptapeptide β-strands (Aβ_17–23_ and Aβ_30–36_ or Aβ_29–35_) connected by two δ-linked ornithine turn units that promote a β-hairpin-like conformation. The peptides also contain an *N*-methyl group on Gly_33_ (Aβm_17–36_) or Ile_32_ (Aβm_17–35_) to attenuate aggregation through the formation of intermolecular hydrogen bonds. These features facilitate the formation of well-defined oligomers that, unlike oligomers of full-length Aβ, are amenable to high-resolution structural characterization through X-ray crystallography.

Aβm_17–36_ is designed to display the side chains of Val_36_, Leu_34_, Ile_32_, and Ala_30_ on the same face of the β-hairpin as the side chains of Leu_17_, Phe_19_, Ala_21_, and Asp_23_ (the LFAD face). The side chains of Met_35_, *N*-methyl-Gly_33_, and Ile_31_, as well as the side chains of Val_18_, Phe_20_, and Glu_22_, are in turn displayed on the opposite face (the VFE face). Aβm_17–35_ is designed to display the side chains of Met_35_, Gly_33_, Ile_31_, and Gly_29_ on the LFAD face and the side chains of Leu_34_, *N*-methyl-Ile_32_, and Ala_30_ on the VFE face. Figure 1 illustrates these differences through the colors of the side chains (green and orange). While both peptides primarily consist of hydrophobic residues, the differences between the faces of Aβm_17–36_ and Aβm_17–35_ lead to the formation of different oligomeric assemblies.

### Assembly by SDS-PAGE

**Figure 2:**
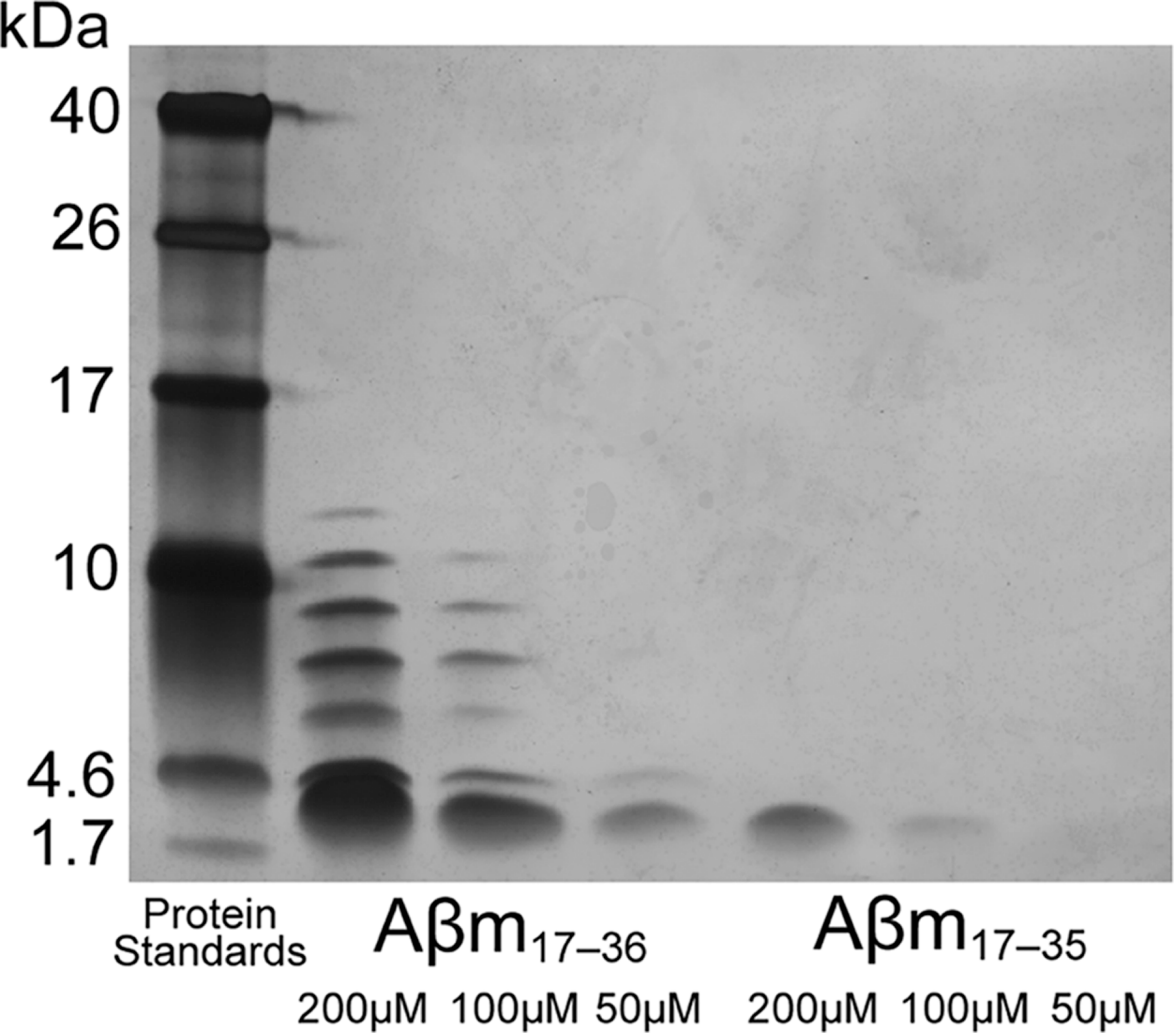
Silver-stained SDS-PAGE gel of Aβm_17–36_ and Aβm_17–35_.

Aβm_17–36_ and Aβm_17–35_ were subjected to sodium dodecyl sulfate-polyacrylamide gel electrophoresis (SDS-PAGE) to evaluate their propensity to oligomerize. When a 200 μM solution of Aβm_17–36_ is subjected to SDS-PAGE and visualized by silver staining, a ladder of seven bands is observed (Figure 2). The bands appear to be evenly separated by about 2 kDa, which is consistent with the molecular weight of the monomer (1.76 kDa). The lowest molecular weight band corresponds to either monomer or dimer, and the subsequent bands correspond to either dimer through heptamer or trimer through octamer. The ladderlike appearance suggests that the oligomers are formed by sequential addition of Aβm_17–36_ monomers. The intensities of the bands indicate that the smallest species (monomer or dimer) predominates and that the oligomers corresponding to the second, fourth, fifth, and sixth bands may be more stable than those corresponding to the third. Fewer bands are observed with decreasing concentration, suggesting that oligomer formation is dependent on concentration. Alternatively, the larger oligomers may still be present at lower concentrations but not abundant enough to reach the sensitivity limit of the silver stain.

To our knowledge, the periodic ladderlike oligomerization exhibited by Aβm_17–36_ has not been observed for other Aβ peptides in SDS-PAGE. Aβ_1–42_ typically exhibits prominent monomer, trimer, and tetramer bands in SDS-PAGE, while Aβ_1–40_ typically exhibits predominantly a monomer band.^28−30^ Ladders of oligomers are observed in SDS-PAGE when Aβ_1–40_ or Aβ_1–42_ are subjected to photoinduced cross-linking of unmodified proteins (PICUP).^28,29^ Our laboratory has also observed a ladder of oligomers upon TCEP treatment of an Aβ_1–42_ peptide containing an intramolecular disulfide bond.^31,32^

In contrast to Aβm_17–36_, Aβm_17–35_ migrates as a single diffuse band at molecular weights consistent with a monomer or dimer in SDS-PAGE (Figure 2). Aβm_17–35_ exhibits fainter bands than Aβm_17–36_ at the same concentrations. Staining with Bio-Rad fluorescent stains Flamingo and Oriole also showed slightly weaker bands for Aβm_17–35_ compared to Aβm_17–36_ (data not shown). The decreased intensity of Aβm_17–35_ with both silver staining and fluorescent stains might reflect a greater propensity of Aβm_17–35_ to diffuse out of the gel during the staining process or lower solubility of Aβm_17–35_ leading to poorer penetration into the gel.

### X-ray Crystallography

**Figure 3:**
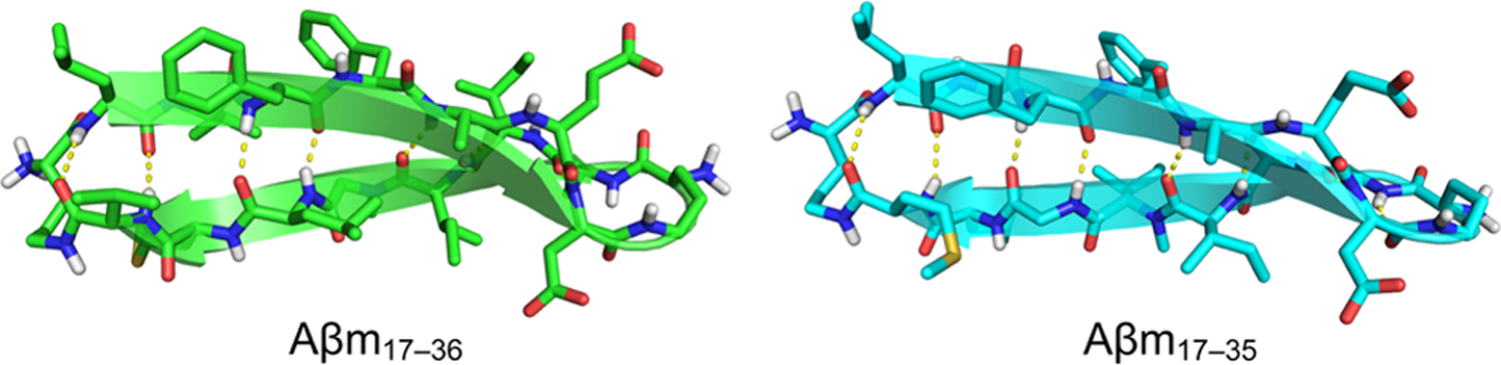
X-ray crystallographic structures of representative monomers of Aβm_17–36_ and Aβm_17–35_ (PDB 8GJD and 8GJC). Aβm_17–36_ contains 16 unique molecules in the asymmetric unit; Aβm_17–35_ contains 2 unique molecules in the asymmetric unit.

Aβm_17–36_ and Aβm_17–35_ both proved to be amenable to structural elucidation by X-ray crystallography (Figure 3). Aβm_17–36_ afforded crystals under conditions used previously for a homologue containing ornithine in place of Met_35_ (HEPES buffer and Jeffamine M-600).^33^ Aβm_17–35_ afforded crystals from a buffer consisting of bicine and Trisma and a mixture of ethylene glycol oligomers. Diffraction data for crystals of Aβm_17–36_ were collected to 2.05 Å in-house on a Rigaku Micromax-007HF X-ray diffractometer equipped with a copper anode. Diffraction data for crystals of Aβm_17–35_ were collected to 1.52 Å on the synchrotron at the Advanced Light Source at Lawrence Berkeley National Laboratory. The X-ray crystallographic phases of Aβm_17–36_ were solved by soaking the crystals in potassium iodide to incorporate iodide ions into the crystal lattice and then performing single-wavelength anomalous diffraction (SAD) phasing. The X-ray crystallographic phases of Aβm_17–35_ were solved using molecular replacement with an all-alanine model of a related β-hairpin peptide as a search model (PDB5W4H).^34^

**Figure 4:**
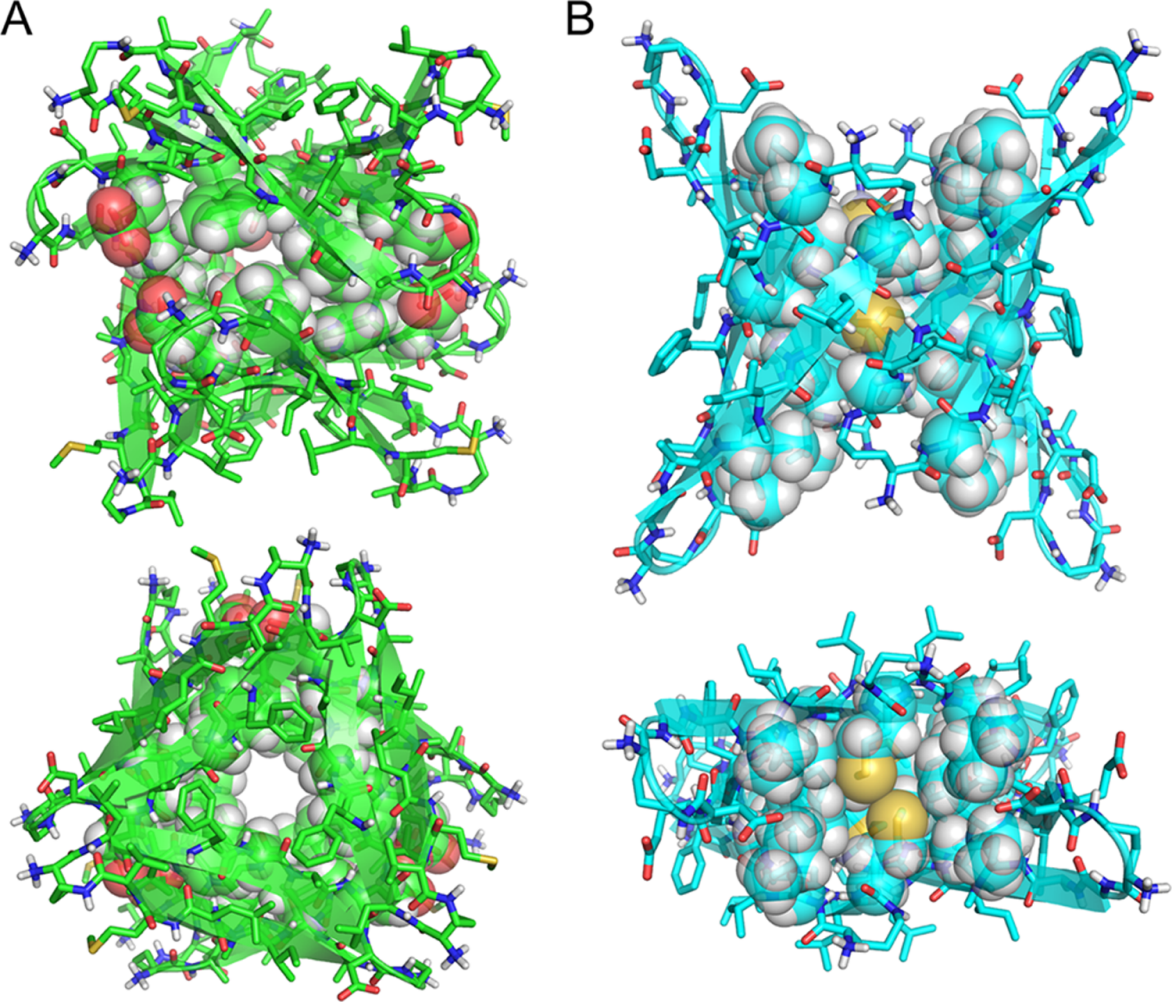
X-ray crystallographic structure of (A) the hexamer formed by Aβm_17–36_ and (B) the tetramer formed by Aβm_17–35_. Side chains of residues in the hydrophobic cores of the assemblies are shown as spheres to illustrate packing. Assemblies are shown from a “side” view (top) and then rotated 90 to show a “top” view (bottom).

Aβm_17–36_ and Aβm_17–35_ form different oligomers in the crystal state. The X-ray crystallographic structure of Aβm_17–36_ contains 16 molecules of Aβm_17–36_ in the asymmetric unit. Each of the molecules folds to form a twisted β-hairpin, and variation between monomers is minimal, consisting mainly of Met_35_ and Leu_34_ rotamers. In the crystal lattice, Aβm_17–36_ assembles to form trimers, which loosely pack into hexamers (Figure 4A). The X-ray crystallographic structure of Aβm_17–35_ contains two molecules of Aβm_17–35_ in the asymmetric unit. Both molecules fold to form twisted β-hairpins, and variation between the two monomers is minimal, consisting mainly of Met_35_ rotamers. The different Met_35_ rotamers likely aid in maximizing hydrophobic packing within the crystal lattice. In the crystal lattice, both molecules of Aβm_17–35_ assemble to form tetrameric β-barrels or cylindrins^35^ (Figure 4B).

**Figure 5:**
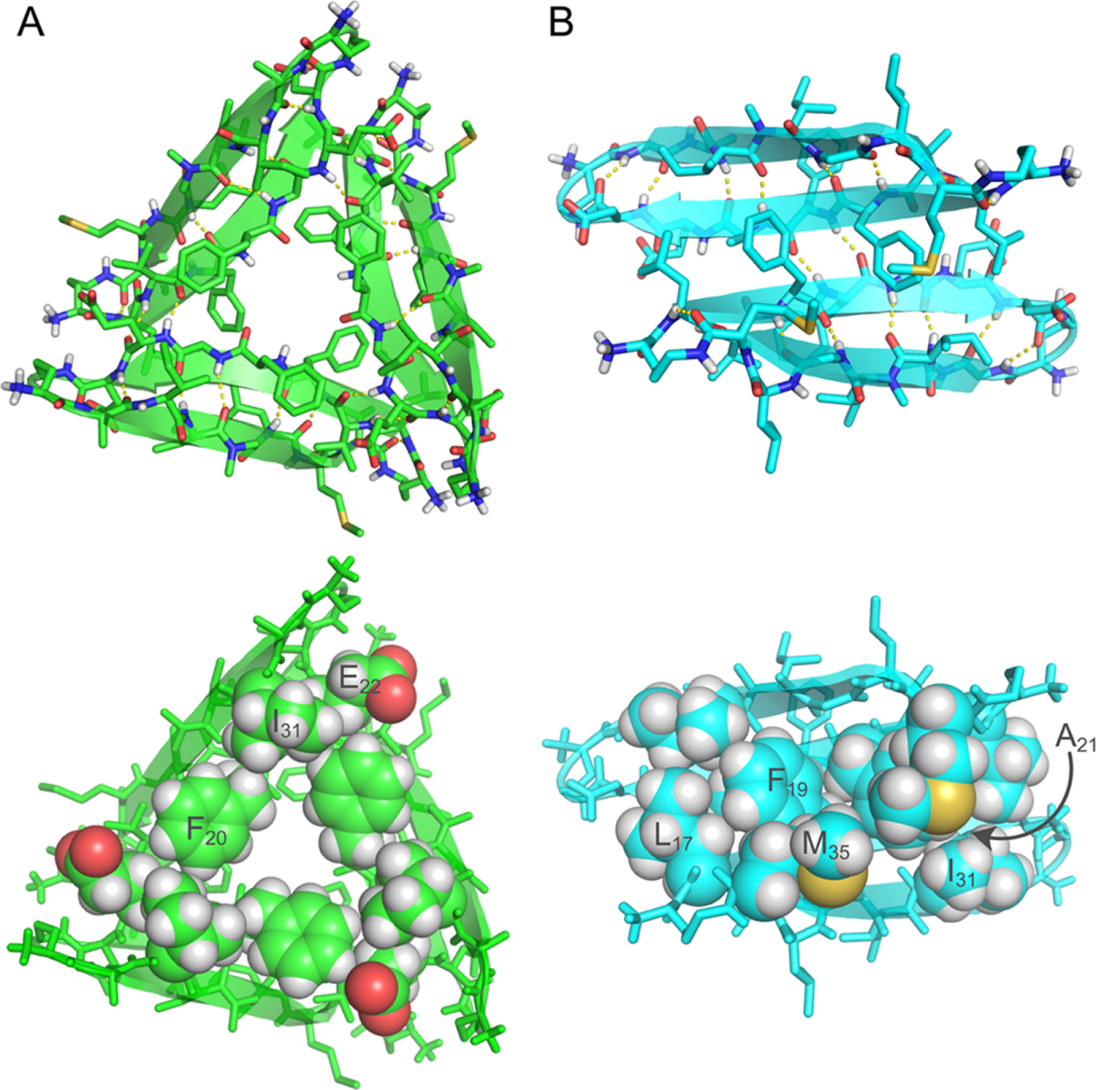
X-ray crystallographic structures of (A) a trimer formed by Aβm_17–36_ and (B) an antiparallel dimer formed by Aβm_17–35_. Hydrogen bonds within the structures are shown by yellow dashed lines (top). The side chains of residues that pack into the hydrophobic core of the hexamer formed by Aβm_17–36_ and the tetramer formed by Aβm_17–35_ are shown as spheres (bottom). These residues are labeled on one monomer within each structure. Ala_21_ in the dimer of Aβm_17–35_ is mostly hidden behind Phe_19_ and Met_35_ of the adjacent molecule of Aβm_17–35_.

The trimers formed by Aβm_17–36_ consist of three β-hairpins in a triangular assembly with a cavity at the center of the triangle (Figure 5A). Phe_20_ and Phe_19_ sit on opposite faces at the center of the trimer surrounding this cavity. The trimer is stabilized by hydrophobic packing of residues on both faces and by intermolecular hydrogen bonding between the backbones of Val_18_ and Glu_22_ at each corner. Two trimers further pack together on the VFE face to form a hexamer. Although the hexamer is not stabilized by hydrogen bonds between the component trimers, it is stabilized by bridging water molecules that hydrogen bond to both trimers. The side chains of residues Phe_20_, Glu_22_, and Ile_31_ form the hydrophobic core of the hexamer, with the carboxyl groups of Glu_22_ sitting at the corners. Although Met_35_ and Gly_33_ also sit on the VFE face of Aβm_17–36_, neither appears to contribute to the packing of the trimer or hexamer.^36^ The remaining side chains sit outside of the hydrophobic core of the hexamer.

The tetramers formed by Aβm_17–35_ consist of four β-hairpins arranged around a central axis to form a β-barrel. The tetramer can be viewed as a dimer of dimers in which two monomers assemble to form an antiparallel dimer (Figure 5B), and two antiparallel dimers further assemble to form the tetramer. The hydrophobic side chains of the LFAD face comprise the core of the tetramer. The side chains of Phe_19_ and Met_35_ lie at the center of the hydrophobic core and are buttressed by the side chains of Leu_17_, Ala_21_, and Ile_31_. The absence of a side chain at Gly_33_ facilitates the tight packing of these hydrophobic side chains in a fashion similar to that previously reported for a cylindrin comprising three β-hairpins from αB crystallin.^35^ The charged residues Glu_22_ and Asp_23_ sit on the solvent-exposed ends of the Aβm_17–35_ cylindrin away from the hydrophobic core. Intermolecular hydrogen bonds between the monomer subunits further stabilize the tetramer. The backbones of the Phe_20_ residues hydrogen bond to each other at the interfaces between monomers within the dimers. The backbones of the Leu_34_ residues hydrogen bond to each other at the interfaces between the dimers.

Although both the hexamer of Aβm_17–36_ and the tetramer of Aβm_17–35_ are stabilized by hydrophobic packing, this packing occurs on opposite faces of the peptides. The hexamer of Aβm_17–36_ packs on the VFE face, even though the LFAD face displays three more hydrophobic side chains (Val_18_, Phe_20_, Ile_31_, and Met_35_ vs Leu_17_, Phe_19_, Ala_21_, Ala_30_, Ile_32_, Leu_34_, and Val_36_). Additional intermolecular contacts in the lattice involving Leu_17_ and Val_36_ may promote packing of the trimers on the less hydrophobic VFE face. The relatively loose packing of the trimers within the hexamer suggests that the trimer is the primary oligomeric building block in the crystal lattice. To this end, the trimer is stabilized by extensive packing of three sets of hydrophobic side chains in its center (Leu_17_, Phe_19_ Phe_20_, Ala_21_, Ile_31_, and Leu_34_).

The Aβm_17–35_ tetramer packs on the LFAD face rather than the VFE face. The two faces each display the same number of hydrophobic side chains (Leu_17_, Phe_19_, Ala_21_, Ile_31_, and Met_35_ vs Val_18_, Phe_20_, Ala_30_, Ile_32_, and Leu_34_). Packing on the LFAD face may result from better self-complementarity of this face, permitting tighter hydrophobic packing. No major hydrophobic interactions are observed between the tetramers in the lattice, despite the exterior of each tetramer presenting multiple hydrophobic side chains. Although the tetramers pack together in the lattice, the packing interactions do not appear to lead to well-defined higher-order assemblies.

The assemblies observed for Aβm_17–36_ and Aβm_17–35_ in the crystal state do not match the assemblies observed in SDS-PAGE. These differences likely result from the different experimental conditions required for SDS-PAGE and crystallization.^53^ SDS-PAGE is run at micromolar concentrations in a pH 6.8 Tris loading buffer containing 2% glycerol. Crystallization occurs at millimolar concentrations at pH 6.75 and pH 8.5 for Aβm_17–36_ and Aβm_17–35_, respectively. The varied behaviors of Aβm_17–36_ and Aβm_17–35_ across different techniques are reminiscent of that of full-length Aβ peptides, for which assembly is highly dependent on experimental conditions.^37−52^ Peptide concentration, the presence of detergents or lipids, pH, and temperature, among other factors, can direct, alter, induce, or inhibit assembly in full-length Aβ peptides.^37−52^

Aβm_17–36_ and Aβm_17–35_ mimic just two of the many conformations that full-length Aβ can form. In this paper, X-ray crystallography and SDS-PAGE demonstrate that the alignment of the β-strands within an Aβ-derived β-hairpin affects how the peptide oligomerizes. The range of assemblies Aβm_17–36_ and Aβm_17–35_ form under different conditions exemplifies how β-hairpin assembly can be affected by environment and provides insight into the factors that drive the oligomerization of Aβ peptides. The current study provides evidence that β-hairpin formation, along with environmental variation, can contribute to Aβ oligomer heterogeneity. While the heterogeneity of Aβ oligomers is still an obstacle to understanding the molecular basis of AD, this study illustrates how small changes in the folding of the component Aβ monomer subunits can have profound effects on Aβ oligomer structure and assembly.

## Materials/Experimental Methods

Peptides Aβm_17–36_ and Aβm_17–35_ were synthesized by procedures analogous to those described previously.^23,53^ SDS-PAGE and silver staining were performed, as described previously.^23^ Procedures detailing the preparation of Aβm_17–36_ and Aβm_17–35_, SDS-PAGE, silver staining, and X-ray crystallography can be found in the Supporting Information.

## Supporting Information Available

Procedures for the synthesis of peptides Aβm_17–36_ and Aβm_17–35_; SDS-PAGE and silver staining; crystallization conditions; and X-ray crystallographic data collection, processing, and refinement can be found in the Supporting Information (PDF)

## Accession Codes

PDB 8GJD PDB 8GJC
