# Correction to “β-Hairpin Alignment Alters Oligomer Formation in Aβ-Derived Peptides”

**DOI:** 10.1021/acs.biochem.4c00037

**Published:** 2024-02-15

**Authors:** Sarah M. Ruttenberg, Adam G. Kreutzer, Nicholas L. Truex, James S. Nowick

Due to an oversight on our part, we did not properly acknowledge the Advanced Light Source Beamline that allowed the X-ray crystallographic structure determination in this manuscript. The Acknowledgments section has been rewritten as below to reflect this oversight.

