# Supplementary material for: β-Hairpin Alignment Alters Oligomer Formation in Aβ-Derived Peptides
Source: Biochemistry. 2024 Jan 1;63(2):212–8. doi: 10.1021/acs.biochem.3c00526 (PMC10795187; doi:10.1021/acs.biochem.3c00526)
Supplement: Supplementary file 1 — bi3c00526_si_001.pdf [file bi3c00526_si_001.pdf]

Supporting Information for:

β-Hairpin Alignment Alters Oligomer Formation in Aβ-Derived Peptides

Sarah M. Ruttenberg, Adam G. Kreutzer, Nicholas L. Truex, James S. Nowick*

Department of Chemistry, University of California, Irvine
Irvine, California 92697-2025, United States

*To whom correspondence should be addressed: jsnowick@uci.edu

Table of Contents

Table S1 – Crystallographic Properties of PDB S2

Materials and Methods S3

General Information S3

Peptide Synthesis S3

SDS-PAGE and silver staining S5

Crystallization Procedures S6

X-ray diffraction data collection, data processing, and structure determination S7

References and Notes S8

Characterization Data S9

Characterization of $Aβm_{17–36}$ S9

Characterization of $Aβm_{17–35}$ S11

Table S1

Peptide	Aβm$_{17–36}$	Aβm$_{17–35}$
PDB ID	8GJD	8GJC
Space Group	R 3 :H	P 32 2 1
a, b, c (Å)	68.1848, 68.1848, 168.564	36.164, 38.164, 32.054
Α, β, λ (°)	90, 90, 120	90, 90, 120
Peptides per Asymmetric Unit	16	2
Wavelength (Å)	1.54	0.998
Resolution (Å)	34.09 - 2.03 (2.103 - 2.03)	23.01 - 1.431 (1.482 - 1.431)
Total Reflections	399917 (18431)	81086 (1686)
Unique Reflections	18832 (1806)	5078 (384)
Multiplicity	21.2 (10.2)	16.0 (4.4)
Completeness (%)	99.49 (96.27)	97.02 (74.46)
Mean I/σ	35.86 (4.81)	24.44 (0.71)
Wilson B factor	23.85	15.0
R_{merge}	0.2206 (0.57)	0.09013 (1.775)
$R_{measure}$	0.2253 (0.5992)	0.09285 (2.026)
$CC_{1/2}$	0.981 (0.899)	0.999 (0.25)
CC^*	0.995 (0.973)	1 (0.633)
R_{work}	0.2465 (0.2797)	0.1545 (0.5371)
R_{free}	0.2901 (0.3380)	0.1789 (0.5666)
Number of Non-Hydrogen Atoms	2180	301
RMS_{bonds}	0.020	0.009
RMS_{angles}	0.81	1.20
Ramachandran Favored (%)	100	100
Outliers (%)	0	0
Clashscore	1.83	12.10
Average B-Factor	26.19	21.14

Materials and Methods[1]

General information

All chemicals were used as received unless otherwise noted. All Fmoc-protected amino acids, coupling agents, triisopropylsilane (TIPS), and 2-chlorotrityl chloride resin was purchased from Chem-Impex. HPLC grad acetonitrile (MeCN) was purchased from Fischer Scientific. Methylene chloride (DCM) was passed through alumina under nitrogen prior to use. Anhydrous, amine-free *N,N*-dimethylformamide (DMF), DIPEA, 2,4,6-collidine, and piperidine were purchased from Alfa Aesar. Trifluoroacetic acid (TFA) and hexafluoroisopropanol (HFIP) were purchased from Oakwood Chemical. Deionized water (18 MΩ) was obtained from a Barnstead NANOpure Diamond water purification system. Analytical reverse-phase HPLC was performed on an Agilent 1260 instrument equipped with a Phenomonex Aeris PEPTIDE 2.6 µm XB-C18 150 mm x 4.6 mm column. Preparative reverse-phase HPLC was performed on a Rainin Dynamax instrument equipped with an Agilent Zorbax 300SB-C18 21.2 mm x 250 mm (7 µm) column. Peptides were first purified on a Biotage® Isolera™ One system equipped with a 25 g Biotage® Sfär Bio C18 – Duo 300 Å 20 µm column, before repurification on the Rainin Dynamax. UV detection (214 nm) was used for analytical and preparative HPLC. HPLC grade acetonitrile and deionized water, each containing 0.1% trifluoroacetic acid (TFA), were used for analytical and preparative reverse-phase HPLC. Matrix-assisted laser desorption/ionization time-of-flight (MALDI-TOF) mass spectrometry was performed on an AB SCIEX TOF/TOF 5800 system with α-cyano-4-hydroxycinnamic acid as the sample matrix. All peptides were prepared and used as the trifluoroacetate salts and were assumed to have one trifluoroacetic acid molecule per amine group on each peptide.

Synthesis of Aβm$_{17–36}$ and Aβm$_{17–35}$

Loading of the resin. 2-Chlorotrityl chloride resin (300 mg, 1.6 mmol/g) was added to a Bio-Rad Poly-Prep chromatography column. The resin was suspended in dry DCM (8 mL) and allowed to swell for 30 min. The solution was drained from the resin and a solution of Boc-Orn(Fmoc)-OH (0.6 equiv, 100 mg, 0.22 mmol) in 6% (v/v) 2,4,6-collidine in dry DCM (8 mL) was added immediately and the suspension was gently agitated for 12 h. The solution was then drained and a mixture of DCM/MeOH/*N,N*-diisopropylethylamine (DIPEA) (17:2:1, 6 mL) was

added immediately. The mixture was gently agitated for 1 h to cap the unreacted 2-chlorotrityl chloride resin sites. The resin was then washed with dry DCM (2x) and dried by passing nitrogen through the vessel. This procedure typically yields 0.12–0.18 mmol of loaded resin (0.4–0.6 mmol/g loading).

Peptide coupling. The Boc-Orn(Fmoc)-2-chlorotrityl resin generated from the previous step was transferred to a solid phase reaction vessel and washed with DMF (5x). The linear peptide was synthesized from the C-terminus to the N-terminus. Each coupling cycle consisted of i. Fmoc-deprotection with 20% (v/v) piperidine in DMF for 5 min, ii. washing with DMF (5x), iii. coupling of the amino acid (0.75 mmol, 5 equiv) in the presence of HCTU (0.675 mmol, 4.5 equiv) and 20% (v/v) 2,4,6-collidine in DMF for 20 min iv. washing with DMF (5x). Special coupling conditions were used for the isoleucine that followed the *N*-methyl-glycine in $A\beta m_{17–36}$ and for the isoleucine that followed the *N*-methyl-isoleucine in $A\beta m_{17–35}$: The isoleucine was double coupled (0.75 mmol, 5 equiv.) and allowed to react for 1 h per coupling with HATU (5 equiv) and HOAt (5 equiv) in 20% (v/v) 2,4,6-collidine in DMF. After coupling of the last amino acid, the terminal Fmoc group was removed with 20% (v/v) piperidine in DMF (5 min). The resin was transferred from the reaction vessel to a Bio-Rad Poly-Prep chromatography column.

Cleavage of the peptide from the resin. The linear peptide was cleaved from the resin by agitating the resin for 45 min with a solution of 1,1,1,3,3,3-hexafluoroisopropanol (HFIP) in DCM (1:4, 8 mL). The suspension was filtered and the filtrate was collected in a 250-mL round-bottomed flask. An additional 8 ml of the HFIP cleavage cocktail was added to the resin and agitated for 30 min. The solution was then filtered into the same flask. The combined filtrates were concentrated by rotary evaporation to give a white solid, which was cyclized without further purification.

Cyclization of the linear peptide. The crude protected linear peptide was dissolved in dry DMF (150 mL). HOBt (114 mg, 0.75 mmol, 5 equiv) and HBTU (317 mg, 0.75 mmol, 5 equiv) were added to the solution. DIPEA (0.33 mL, 1.8 mmol, 12 equiv) was added to the solution and the mixture was stirred under nitrogen for 48 h. The mixture was by rotary evaporation to afford the crude protected cyclic peptide. Cyclized peptides were further dried by vacuum pump.

Global deprotection and Ether Precipitations. The protected cyclic peptide was dissolved in TFA/triisopropylsilane (TIPS)/H_2O (18:1:1, 20 mL) in a 250-mL round-bottomed flask

equipped with a nitrogen-inlet adaptor. The solution was stirred for 1.5 h. The reaction mixture was then separated evenly into two 50 ml conical tubes. 30 ml of ice-cold diethyl ether was added to each tube and left to sit on ice for 15 minutes. The 50-ml conical tubes were centrifuged (2000 x g) for 10 min to pellet the crude peptide. The supernatant was removed, and the pellets were redissolved in 5 ml of MeCN. The MeCN peptide solutions were combined in a 250 ml round-bottom flask concentrated by rotary evaporation to afford the crude cyclic peptide. The crude cyclic peptide was immediately subjected to purification by reverse-phase HPLC (RP-HPLC), as described below.

Reverse-phase HPLC purification. The peptide was dissolved in H_2O and MeCN (4:1, 10 mL), and purified on a Biotage® Isolera™ One system equipped with a 25 g Biotage® Sfär Bio C18 – Duo 300 Å 20 μm column using a MeCN (15%-45%) gradient in H_2O. Fractions were analyzed by MALDI-TOF and analytical HPLC. Fractions containing the desired peptide were combined in a 250 ml round-bottom flask and concentrated by rotary evaporation. The peptide was then redissolved in H_2O and MeCN (4:1, 10 mL), filtered through a 0.2 μm syringe filter, and purified by RP-HPLC on a Rainin Dynamax instrument equipped with an Agilent Zorbax 300SB-C18 21.2 mm x 250 mm (7 μm) column (gradient elution with 20–45% MeCN in H_2O over 70 min). Fractions were analyzed by MALDI-TOF and analytical HPLC. Pure fractions were concentrated by rotary evaporation and lyophilized. Typical syntheses yielded ~30 mg of the peptide as the TFA salt.

SDS-PAGE and silver staining

Solutions of $A\beta m_{17-36}$ and $A\beta m_{17-35}$ were prepared gravimetrically by dissolving lyophilized peptide in the appropriate amount of 18 MΩ deionized water to achieve a 10 mg/ml stock. Stock solutions of all peptides were diluted with 18 MΩ deionized water to create 400 μM, 200 μM, and 100 μM sample solutions. 1 μl of 6X SDS-PAGE sample loading buffer (G Biosciences) and 2 μl 18 MΩ deionized water was added per 3 μl of sample solution to create working solutions. 5 μl aliquots of each working solution were run on a 16.5% polyacrylamide Mini-PROTEAN® Tris/Tricine Precast Gel from Bio-Rad Laboratories.[2] Reagents for Tricine SDS-PAGE were prepared and used according to recipes and procedures detailed in the Mini-PROTEAN Precast Gels Instruction Manual and Application Guide (2011) from Bio-Rad Laboratories.[2] The gel was run at a constant 100 V for approximately 2 hours. The migration of

$A\beta m_{17–36}$ and $A\beta m_{17–35}$ was compared with a molecular weight protein ladder (SpectraTM Multicolor Low Range Protein Ladder, ThermoFisher Scientific, catalog #: 26628).

Staining with silver nitrate was used to visualize $A\beta m_{17–36}$ and $A\beta m_{17–35}$ in the SDS-PAGE gel. Reagents for silver staining were prepared according to procedures detailed in Simpson, R. J. CSH Protoc. 2007.[3] [The sodium thiosulfate solution, silver nitrate solution, and developing solution were prepared fresh each time silver staining was performed]. Briefly, the gel was removed from the casting glass and rocked in fixing solution (50% (v/v) MeOH and 5% (v/v) acetic acid in deionized water) for 20 min. Next, the fixing solution was discarded and the gel was rocked in 50% (v/v) aqueous MeOH for 10 min. Next, the 50% methanol was discarded and the gel was rocked in deionized water for 10 min. Next, the water was discarded and the gel was rocked in 0.02% (w/v) sodium thiosulfate in deionized water for 1 min. The sodium thiosulfate was discarded and the gel was rinsed with deionized water for 1 min (2X). After the last rinse, the gel was submerged in chilled 0.1% (w/v) silver nitrate in deionized water and rocked at 4 °C for 20 min. Next, the silver nitrate solution was discarded and the gel was rinsed with deionized water for 1 min (2X). To develop the gel, the gel was incubated in developing solution (2% (w/v) sodium carbonate, 0.04% (w/v) formaldehyde until the desired intensity of staining was reached (~1–3 min). When the desired intensity of staining was reached, the development was stopped by discarding the developing solution and submerging the gel in 5% aqueous acetic acid.

Crystallization conditions for $A\beta m_{17–36}$ and $A\beta m_{17–35}$

Crystallization conditions for $A\beta m_{17–36}$ were determined using a 4x6 matrix Hampton VDX 24-well plate. The conditions mimicked the optimization screen previously used for a homologue of $A\beta m_{17–36}$ that contained ornithine in place of Met_{35} (PDB 4NTR) which previously crystallized in HEPES with Jeffamine M-600.[4] The HEPES buffer pH was varied in each row in increments of 0.5 pH units (6.5, 7.0, 7.5, and 8.0) and the Jeffamine concentration in each column in increments of 2% (24%, 26%, 28%, 30%, 32%, 34%). The first well in the 4x6 matrix for $A\beta m_{17–36}$ was prepared by combined 100 μL of 1 M HEPES buffer at pH 6.5, 480 μL of 50% v/v aqueous Jeffamine M-600 pH 7.0, and 420 μL of deionized water. The other wells were prepared in analogous fashion, by combining 100 μL of HEPES buffer of varying pH, Jeffamine in varying amounts, and deionized water for a total volume of 1 mL in each well.

Three hanging-drops were prepared per borosilicate glass slide by combining a solution of peptide 2 or peptide 4 (10 mg/mL in deionized water) and the well solution in the following amounts: 1 μL:1 μL, 2 μL:1 μL, and 1 μL:2 μL. Crystallization conditions for $A\beta m_{17–36}$ were further optimized using the same method. The HEPES buffer pH was varied in each row in increments of 0.2 pH units (6.1, 6.3, 6.5, and 6.7) and the Jeffamine concentration in each column in increments of 1% (21%, 22%, 23%, 24%, 25%, 26%). Crystals suitable for diffraction grew in most of the pH increments and most of the Jeffamine concentrations.

Initial crystallization conditions for $A\beta m_{17–35}$ were determined using the hanging nanodrop vapor-diffusion method. Crystallization conditions were screened using six crystallization kits in a 96-well plate format (Hampton Index, PEG/Ion, and Crystal Screen; Molecular Dynamics Morpheus, MemPlus/MemSys, PGA). Three 150 nL hanging drops that differed in the ratio of peptide to well solution were made per condition in each 96-well plate for a total of 1728 experiments. Hanging drops were made by combining an appropriate volume of $A\beta m_{17–36}$ or $A\beta m_{17–35}$ (10 mg/mL in deionized water) with an appropriate volume of well solution to create three 150 nL hanging drops with 1:1, 1:2, and 2:1 peptide:well solution. The hanging drops were made using a TTP LabTech Mosquito nanodisperse instrument. Crystals of $A\beta m_{17–35}$ suitable for diffraction grew in a Molecular Dynamics' Morpheus solution containing 0.12M ethylene glycols mix, 0.1M Buffer System 3 pH 8.5, and 37.5% v/v precipitant mix 4. Molecular Dynamics' ethylene glycols mix consists of 0.3 M diethylene glycol, 0.3 M triethylene glycol, 0.3 M tetraethylene glycol, 0.3 M penta(ethylene glycol). Molecular Dynamic's Buffer System 3 consists of 1 M BICINE and 1 M Trisma Base. Molecular Dynamics precipitant mix 4 consists of 25 % w/v hexaethylene glycol, 25 % w/v poly(ethylene glycol) 1000, and 25 % w/v poly(ethylene glycol) 3350.[5]

X-ray diffraction data collection, data processing, and structure determination for peptides $A\beta m_{17–36}$ and $A\beta m_{17–35}$.

Crystals were harvested with a nylon loop attached to a copper or steel pin and flash frozen in liquid nitrogen prior to data collection. $A\beta m_{17–36}$ was soaked in a 1:1 mixture of well solution and 1 M potassium iodide for approximately 5 minutes prior to flash freezing to incorporate iodide ions into the crystal lattice.

Diffraction data for Aβ$m_{17–36}$was collected on a Rigaku Micromax-007HF X-ray diffractometer with a rotating copper anode at 1.54 Å wavelength with 0.5° oscillation. Diffraction data were collected using CrystalClear. Diffraction data were scaled and merged using XDS.[6] Coordinates for the anomalous signals were determined by HySS in the Phenix software suite 1.10.1.7.[7] Electron density maps were generated using single-wavelength anomalous diffraction (SAD) using the anomalous signal from iodine ions incorporated into the crystal lattice from soaking the crystals in KI prior to data collection. The electron density map for Aβ$m_{17–36}$ was generated using anomalous coordinates determined by HySS as initial positions in Autosol.

Diffraction data for Aβ$m_{17–35}$ was collected at the Advanced Light Source at Lawrence Berkeley National Laboratory with a synchrotron source at 0.998-Å wavelength. Data for Aβ$m_{17–35}$ suitable for refinement at 1.431 Å were obtained from the synchrotron. Diffraction data were scaled and merged using XDS.[6] The electron density map for Aβ$m_{17–35}$ was generated by molecular replacement using the coordinates from an all-alanine model of a monomeric peptide from the structure with PDB accession ID 5W4H.[1,8] Molecular manipulation of the Aβ$m_{17–36}$ and Aβ$m_{17–35}$ models was performed with Coot.[6] Coordinates for Aβ$m_{17–36}$ and Aβ$m_{17–35}$ were refined with phenix.refine.

References and Notes

1. These procedures follow closely those that our laboratory has previously published. The procedures in this section are adapted from and in some cases taken verbatim from Kreutzer, A. G., Spencer, R. K., McKnelly, K. J., Yoo, S., Hamza, I. L., Salveson, P. J., Nowick, J. S. (2017). A hexamer of a peptide derived from Aβ$_{16–36}$. *Biochemistry*, *56*(45), 6061–6071. 10.1021/acs.biochem.7b00831
2. BioRad. (n.d.). *Mini-PROTEAN® tris/tricine precast gels*. BioRad. Retrieved March 7, 2023, from https://www.bio-rad.com/en-us/product/mini-protean-tris-tricine-precast-gels?ID=N3GRYEKG4
3. Simpson, R. J. (2007). Staining proteins in gels with silver nitrate. *Cold Spring Harbor Protocols*, *2007*(7). https://doi.org/10.1101/pdb.prot4727
4. Kreutzer, A. G., Yoo, S., Spencer, R. K., Nowick, J. S. (2017). Stabilization, Assembly, and Toxicity of Trimers Derived from Aβ. *Journal of the American Chemical Society*. *139* (2), 966–975. 10.1021/jacs.6b11748.
5. Calibre Scientific. (n.d.). Moleculardimensions.com. Retrieved March 7, 2023, from https://www.moleculardimensions.com/products/morpheus-mixes

6. Emsley, P., Lohkamp, B., Scott, W. G., & Cowtan, K. (2010). Features and development of *coot*. *Acta Crystallographica Section D Biological Crystallography*, *66*(4), 486–501. https://doi.org/10.1107/s0907444910007493
7. Kabsch, W. (2010). *xds*. *Acta Crystallographica Section D Biological Crystallography*, *66*(2), 125–132. https://doi.org/10.1107/s0907444909047337
8. Adams, P. D., Afonine, P. V., Bunkóczi, G., Chen, V. B., Davis, I. W., Echols, N., Headd, J. J., Hung, L.-W., Kapral, G. J., Grosse-Kunstleve, R. W., McCoy, A. J., Moriarty, N. W., Oeffner, R., Read, R. J., Richardson, D. C., Richardson, J. S., Terwilliger, T. C., & Zwart, P. H. (2010). *phenix*: A comprehensive python-based system for Macromolecular Structure Solution. *Acta Crystallographica Section D Biological Crystallography*, *66*(2), 213–221. https://doi.org/10.1107/s0907444909052925

Characterization Data

Characterization of Aβm$_{17–36}$

Analytical HPLC trace of Aβm$_{17–36}$

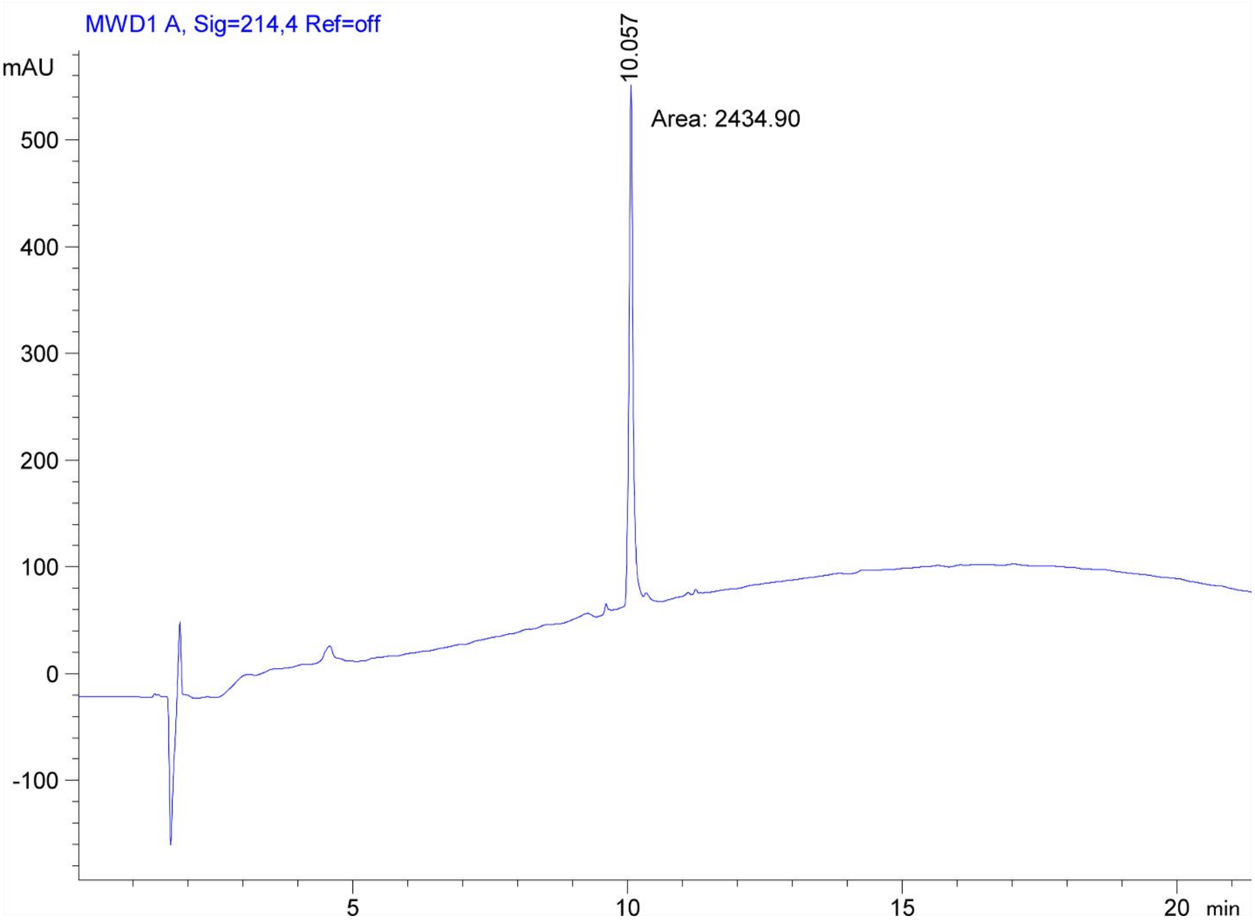

High-resolution mass spectrometry of Aβ$m_{17–36}$

Calculated Mass for Aβ$m_{17–36}$: 1760.99 g/mol
$[M+1H^{+}]^{+}$ = 1762.03
$[M+2H^{+}]^{2+}$ = 881.42

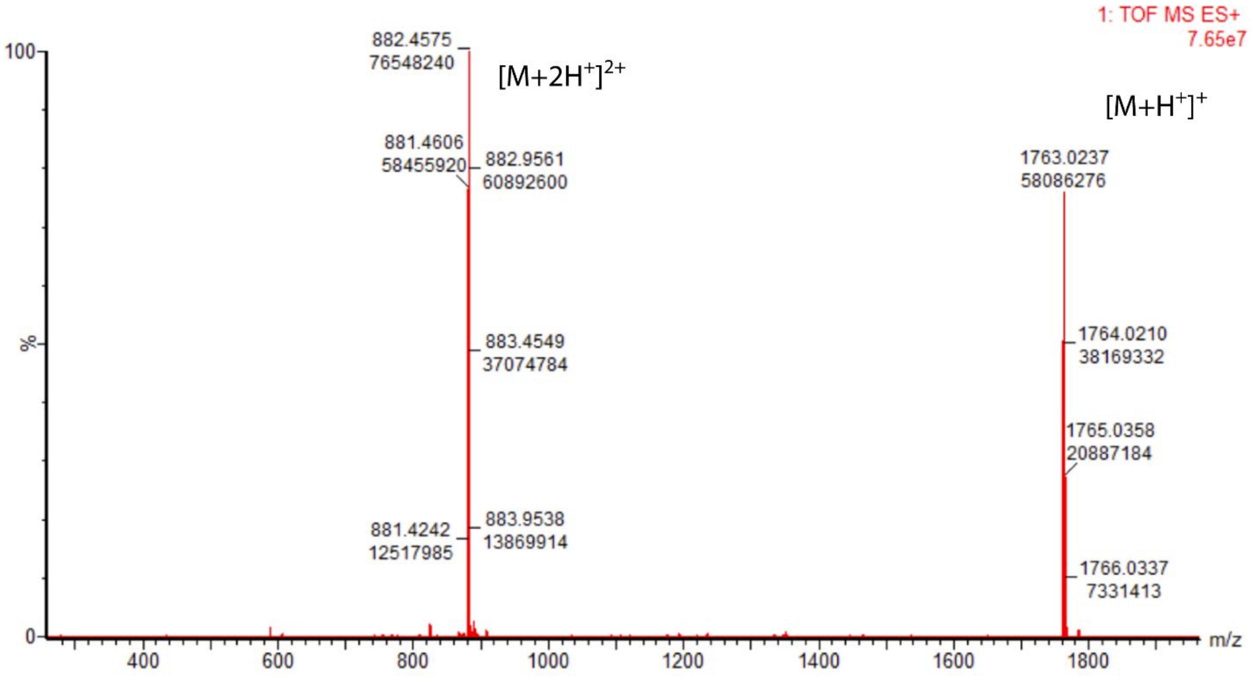

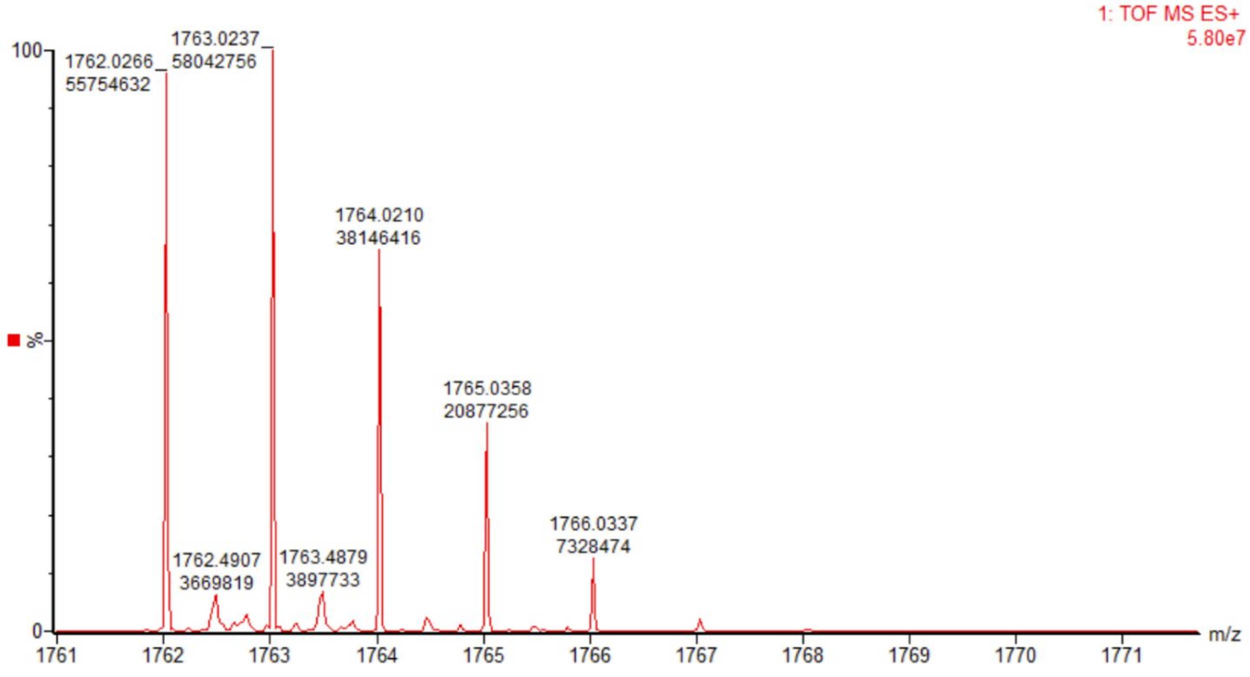

Characterization of Aβm$_{17–35}$

Analytical HPLC trace of Aβm$_{17–35}$

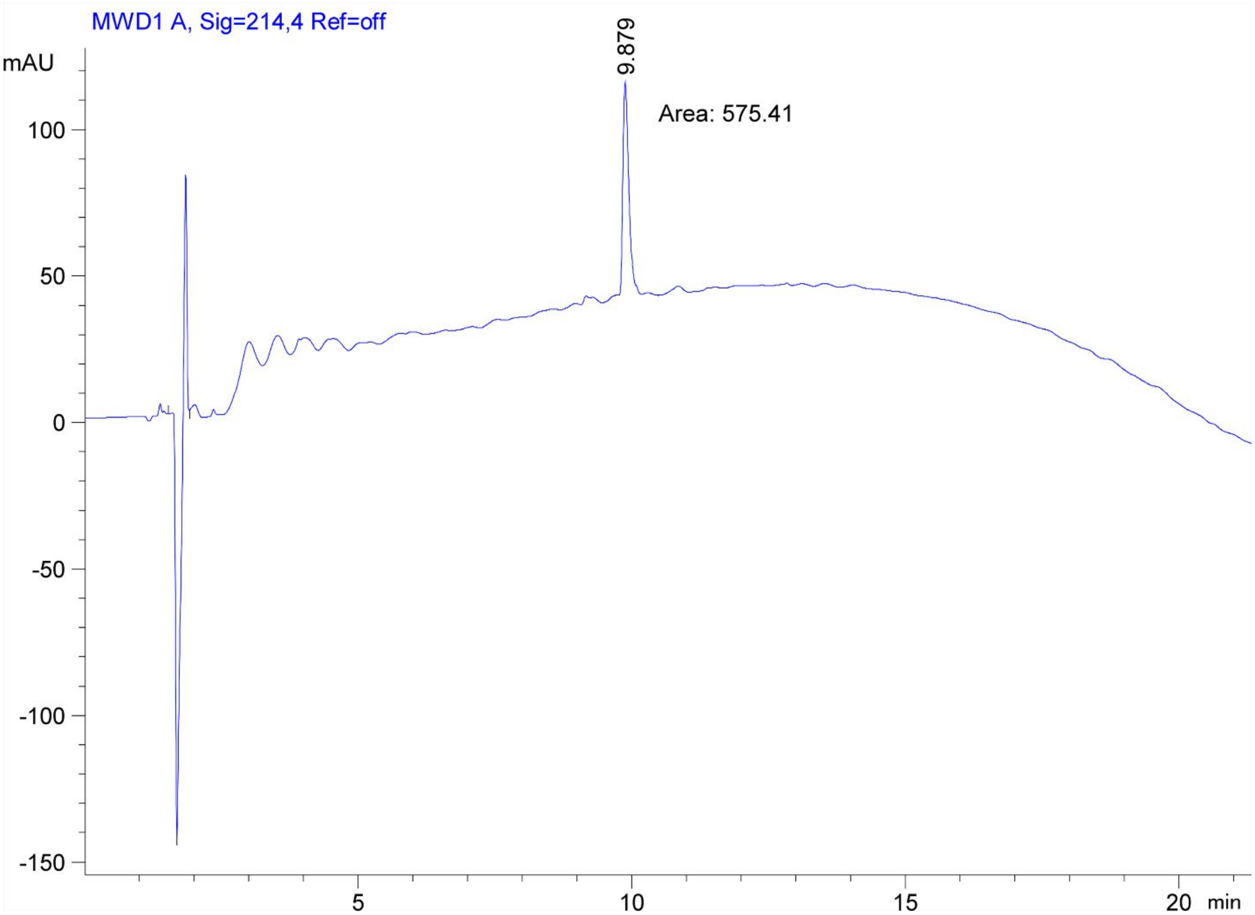

High-resolution mass spectrometry of Aβm$_{17–35}$

Calculated Mass for Aβm$_{17–36}$: 1718.94 g/mol
$[M+1H^{+}]^{+}$ = 1719.96
$[M+2H^{+}]^{2+}$ = 860.43
$[M+3H^{+}]3^{+}$ = 574.31

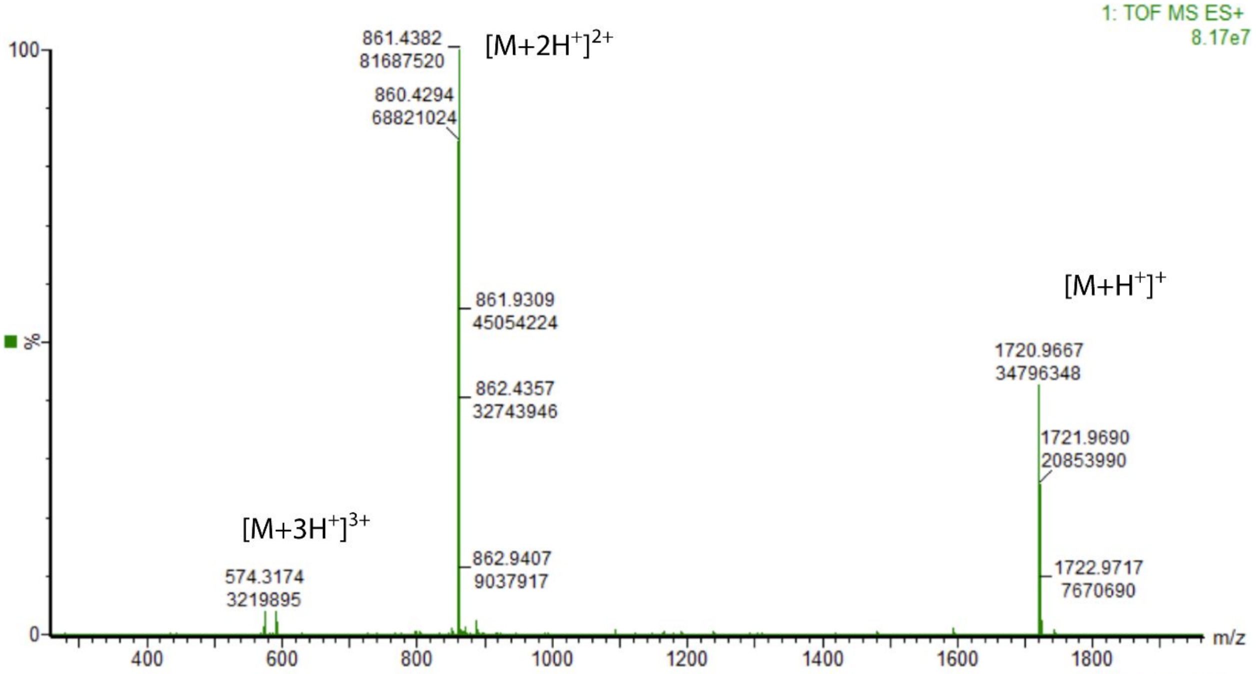
1: TOF MS ES+
8.17e7
[M+2H+]2+
861.4382
81687520
860.4294
68821024
861.9309
45054224
862.4357
32743946
862.9407
9037917
[M+3H+]3+
574.3174
3219895
[M+H+]+
1720.9667
34796348
1721.9690
20853990
1722.9717
7670690
100
%
0
400
600
800
1000
1200
1400
1600
1800
m/z

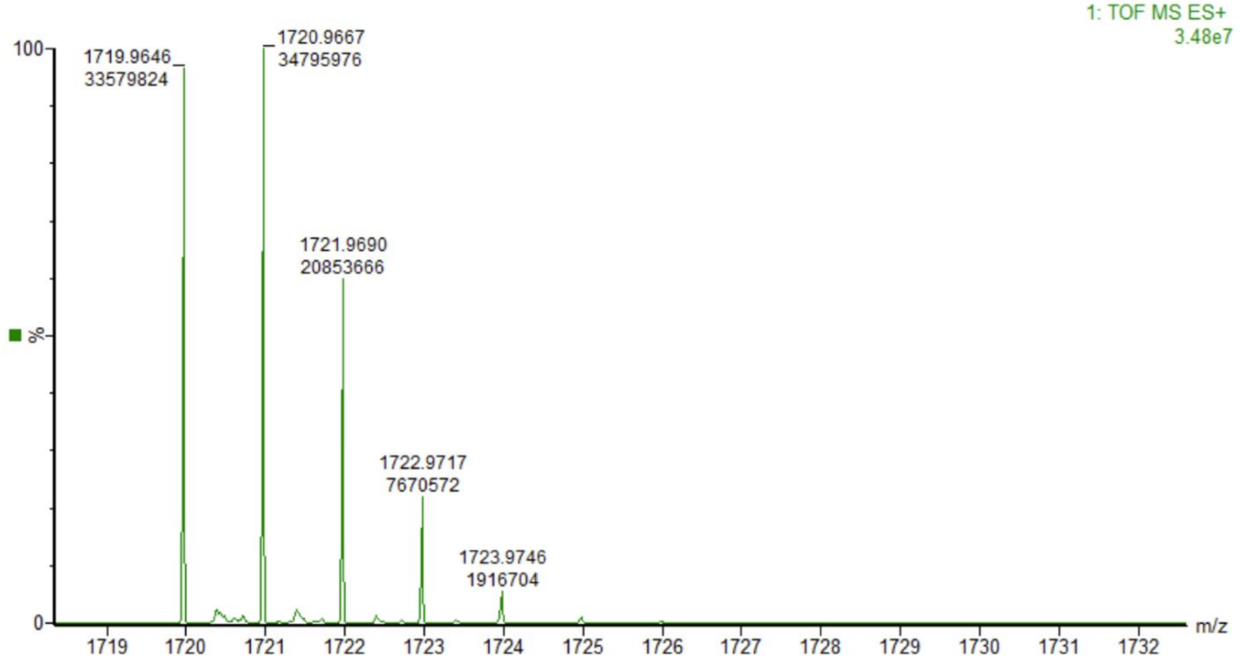
1: TOF MS ES+
3.48e7
1719.9646
33579824
1720.9667
34795976
1721.9690
20853666
1722.9717
7670572
1723.9746
1916704
100
%
0
1719
1720
1721
1722
1723
1724
1725
1726
1727
1728
1729
1730
1731
1732
m/z
